# Late-stage diversification of bacterial natural products through biocatalysis

**DOI:** 10.3389/fbioe.2024.1351583

**Published:** 2024-05-14

**Authors:** Jelena Lazic, Vuk Filipovic, Lena Pantelic, Jelena Milovanovic, Sandra Vojnovic, Jasmina Nikodinovic-Runic

**Affiliations:** Institute of Molecular Genetics and Genetic Engineering, University of Belgrade, Belgrade, Serbia

**Keywords:** bacterial natural products, bioactive molecules, late-stage modification, biotransformation, biocatalysis, enzymatic diversification

## Abstract

Bacterial natural products (BNPs) are very important sources of leads for drug development and chemical novelty. The possibility to perform late-stage diversification of BNPs using biocatalysis is an attractive alternative route other than total chemical synthesis or metal complexation reactions. Although biocatalysis is gaining popularity as a green chemistry methodology, a vast majority of orphan sequenced genomic data related to metabolic pathways for BNP biosynthesis and its tailoring enzymes are underexplored. In this review, we report a systematic overview of biotransformations of 21 molecules, which include derivatization by halogenation, esterification, reduction, oxidation, alkylation and nitration reactions, as well as degradation products as their sub-derivatives. These BNPs were grouped based on their biological activities into antibacterial (5), antifungal (5), anticancer (5), immunosuppressive (2) and *quorum* sensing modulating (4) compounds. This study summarized 73 derivatives and 16 degradation sub-derivatives originating from 12 BNPs. The highest number of biocatalytic reactions was observed for drugs that are already in clinical use: 28 reactions for the antibacterial drug vancomycin, followed by 18 reactions reported for the immunosuppressive drug rapamycin. The most common biocatalysts include oxidoreductases, transferases, lipases, isomerases and haloperoxidases. This review highlights biocatalytic routes for the late-stage diversification reactions of BNPs, which potentially help to recognize the structural optimizations of bioactive scaffolds for the generation of new biomolecules, eventually leading to drug development.

## 1 Introduction

The metabolic diversity of microorganisms, marine organisms, plants, insects and animals results in the production of diverse secondary metabolites, commonly referred to as natural products (Guo, 2017). Bacterial natural products (BNPs) represent a substantially large class of biomolecules which have often played a pivotal role in the treatment of various human pathological conditions throughout our history (Atanasov et al., 2021; Huang et al., 2021). However, exploitation of BNPs from the native host microorganisms is always challenging and only 1% of all bacterial species could be cultured, leaving a vast majority of the species utilizable in perspective (Atanasov et al., 2021). Nevertheless, bacterial strains once deemed “uncultured” are successfully cultivated under laboratory conditions using the iChip device, which was employed in the discovery of antibiotic teixobactin from *Eleftheria terrae* (Piddock, 2015), but it cannot be universal for many other microbial species.

The concept of late-stage diversification is a unique and economical method of regioselective functionalization aimed towards diversity-oriented synthesis and optimization of lead drug candidates with intricate structures. One organic pharmacophore of a starting drug candidate can be effectively utilized to direct various functionalization reactions (C–H activations, O– and N–functionalizations), which can afford different analogs (Dai et al., 2011; Wencel-Delord and Glorius, 2013; Cernak et al., 2016).

Nature’s catalysts - enzymes - have been enclosed in the chemical synthesis toolbox for their remarkable properties and high selectivity under mild conditions, which are worthwhile features in the late-stage diversification of complex molecular structures (Riva, 2001). The strategies of enzymatic reactions in endgame transformations, *i.e.*, obtaining the final product from the late-stage intermediate, have gained recent interest from organic chemists (Kaspar and Schallmey, 2022). Microorganisms and their enzymes were exploited for the enzymatic deracemization, reduction of carbonyl groups, double bonds or carboxylic acids, epoxidation, hydrolysis, decarboxylation, hydroxylations and others, demonstrating a wide scope of reactions and an open space for a large-scale green process development (Wohlgemuth, 2010; Birolli et al., 2019; Hauer, 2020; Liu and Kokare, 2023). Biocatalysis, the use of biological systems (whole cells) or their parts (cell extracts or purified enzymes) to catalyze organic chemical reactions, is a powerful method for the generation of novel derivatives of different drug leads. It offers the possibility for structure manipulation to make natural product leads more drug-like, enables structure-activity relationship (SAR) studies, and can simplify multistep chemical synthesis, thus reducing the costs, side reactions and toxic by-products (Riva, 2001; Guo, 2017; Atanasov et al., 2021).

**FIGURE 1:**
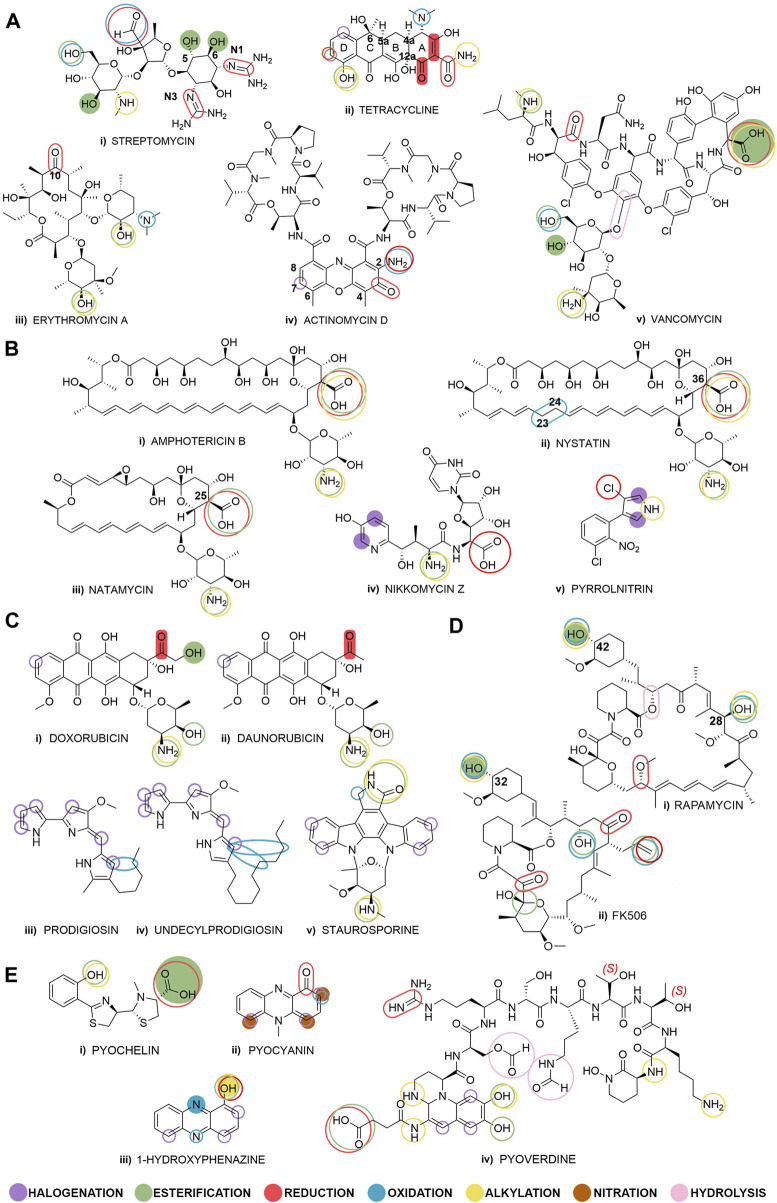
Selected bacterial natural products grouped by their biological activity: **(Ai–v)** antibiotics, **(Bi–v)** antifungals, **(Ci–v)** anticancer compounds, **(Di–ii)** immunosuppressants, and **(Ei–iv)** *quorum* sensing modulators. Fully colored shapes denote known and described diversification reactions achieved through biocatalysis and shown in this review paper, and empty shapes denote potential places for biocatalytic transformations. The overlapping shapes indicate that multiple diversification reactions are possible on a specific functional group.

This review presents late-stage diversifications on a selection of 21 BNP molecules, which are organized by their biological activity into five groups (Figure 1), thus giving an overview of previously reported biocatalytic reactions. This should display possibilities and provide guidance for using biocatalysis in the field of BNPs. A recent review on BNPs grouped according to their biological activity was an inspiration for this classification (Pham et al., 2019). All molecules covered in this study, are secondary metabolites from either *Streptomyces* spp. (Harir et al., 2018; Alam et al., 2022) or *Pseudomonas* spp. (Leisinger and Margraff, 1979; Budzikiewicz, 1993) and were selected both for their array of structures and biological activities, as well as our previous work done with some of these BNPs (Stankovic et al., 2012; Mojicevic et al., 2020; Lazic et al., 2022; Stevanovic et al., 2022; Pantelic et al., 2023).

Reviewing the literature for the period of last 10 years using SciFinder (performed in October 2023) revealed that publications containing the phrase ‘bacterial natural products’ in the title count 95 publications, ‘late-stage modification’ count 240 publications, while publications with the word ‘biocatalysis’ are vast and count 1217 results. However, no results were found for the combination of all three phrases in the title, while linking each of the two phrases yielded only 4-7 relevant results. These literature search results showed that relevant review papers focused either on biocatalytic late-stage modification of non-bacterial natural products (or bacterial products not covered in this review) or on chemical modifications of natural products (including those of bacterial origin) (Chen et al., 2015; Li and Lou, 2018; Hong et al., 2020; Fessner et al., 2022). A database search was conducted on the SciFinder chemical reaction database (performed in November 2023), examining each of the BNPs outlined in this review (Figure 1). Results show that the top 3 molecules used as starting compounds are doxorubicin, rapamycin and vancomycin, whose utilization for derivatization reactions has been described in a large number of publications (957, 755 and 482 publications, respectively), including both journals and patents. More than 100 reactions per molecule were reported for amphotericin B (311), erythromycin A (266), daunorubicin (234), FK506 (198) and staurosporine (124). However, despite numerous publications, there are only a few that describe late-stage biocatalysis reactions and they are described in this review. This suggests general scientific interest in late-stage modifications of these molecules, but also shows research focus has been on already approved and clinically used drugs which are widely available. Fewer than 100 reactions per molecule were reported for tetracycline (88), nystatin (49), 1-hydroxyphenazine (41), natamycin (38) and streptomycin (30). Finally, a total of only 23 studies in journals and five patents have been published for pyrrolnitrin (9), actinomycin D (7), prodigiosin (5), pyocyanin (4), pyochelin (2), and nikkomycin Z (1) combined together, while for undecylprodigiosin there are no published data on late-stage modifications. These results show a vast unexamined research space for the study of biocatalytic diversification of these BNPs.

## 2 Antibacterial compounds

During the “golden age” of antibiotics discovery, more than 20 classes of antibiotics were determined and various classification schemes of antibiotics were proposed based on their origin, activity type or mode of action. We categorized antibiotics according to their chemical structure and have described notable examples from the classes of aminoglycosides, tetracyclines, macrolides and peptide antibiotics (Figure 1A) (Vardanyan and Hruby, 2016).

### 2.1 Streptomycin

In the class of aminoglycoside antibiotics, streptomycin (Figure 1Ai) was the first to be discovered in 1944, and it was also the first antibiotic remedy for tuberculosis. Streptomycin demonstrates effectiveness against both Gram-positive and Gram-negative bacteria and has shown remarkable efficacy in treating numerous infectious diseases. Nevertheless, its primary and most significant application continues to be treatment for tuberculosis (Jospe-Kaufman et al., 2020). Streptomycin is obtained from the culture of *Streptomyces griseus* (Becker and Cooper, 2013), and soon after the discovery of the first aminoglycoside antibiotics, it became evident that even a small structural difference can significantly affect its toxicity, without affecting its antibacterial activity (Jospe-Kaufman et al., 2020). Streptomycin is relatively stable under neutral conditions, but it is easily decomposed in acidic and alkaline environments due to the hydrolysis of the ether bonds, thus making small and precise late-stage structural changes difficult (Shen et al., 2017). The most common chemical used for the transformation of streptomycin is sodium cyanoborohydride applied for the alkylation of the secondary amino group of the sugar moiety, as well as for the reduction of the aldehyde group to a primary amine.

Only a few examples of biocatalytic modification of streptomycin are known and were all carried out on a milligram scale. Extracts of post-exponential phase from mycelia of *Streptomyces bikiniensis* ATCC 11062 containing streptidine kinase could catalyze the phosphorylation of the –OH group in positions C-5 or C-6 (position not determined, Figure 1Ai) of the streptidine moiety with adenosine-5′-triphosphate (Miller and Walker, 1969). 6-*O*-Adenylated streptomycin could be obtained by whole cell biocatalysts using *Bacillus subtilis* (Supplementary Table S1, row i) (O'Hara et al., 1988). Both of these derivatives were obtained as results of an investigation of the biosynthetic pathway in streptomycin-producing *Streptomyces* spp. There are vast unexplored possibilities for the biocatalytic diversification of streptomycin, including the reduction of imine groups at positions N1 and N3 using imine reductase and esterification of free hydroxyl groups by lipases.

### 2.2 Tetracycline

Another example of secondary metabolites from soil bacteria belonging to the phylum *Actinobacteria* are tetracyclines, first reported in the late 1940s. Their introduction in therapy began in 1948 with chlortetracycline, produced by *Streptomyces aureofaciens* (Figure 1Aii). Tetracycline was subsequently synthesized through a semi-synthetic process involving the catalytic hydrogenation of chlortetracycline (Vardanyan and Hruby, 2016). From a chemical perspective, tetracyclines belong to the polyketide subclass. They include a linear fused tetracycline nucleus consisting of rings designated as A, B, C and D and there are naturally occurring stereochemical configurations at the 4a, 5a, 6 and 12a positions, including the 4-dimethylamino group, to which various functional groups could be attached (Figure 1Aii). Various chemical reactions of tetracycline are known, including the syntheses of a series of new tetracycline derivatives in the Mannich reaction (Joshi et al., 2007; Sriram et al., 2007), but the systematization of most commonly used catalysts was not possible because of their structural divergence and reaction outcomes. The existence of a variety of tetracyclines, such as chlortetracycline, tetracycline, oxytetracycline and demeclocycline, suggests the involvement of various tailoring enzymes present within the producing *Streptomyces* strains (Lešnik et al., 2015).

The study by Shang et al. is a rare example of the identification of late-stage transformation products, where the authors isolated different metabolites by preparative high-performance liquid chromatography (HPLC) and clearly assigned the molecular structures by detailed spectroscopic analysis (Shang et al., 2016). Namely, when exposed to solid agar plate cultivations of the marine-derived fungi *Paecilomyces* sp., tetracyclin, minocycline and chlortetracycline are quantitatively transformed to seco-cyclines (Supplementary Table S1, row ii), while oxytetracycline and doxycycline are transformed to hemi-cyclines, with a complete loss of antibiotic properties (Supplementary Table S1, row iii). This study demonstrated that fungi could degrade tetracyclines *in situ* and remove them from the environment.

Crude lignin peroxidase (Wen et al., 2009) or crude manganese peroxidase (Wen et al., 2010) produced by *Phanerochaete chrysosporium* were also both used to degrade tetracycline and oxytetracycline *in vitro* and the degradation rates were up to 95% in 5 min. In more research investigations, the application of fungal cultures or their purified enzymes led to a reduction in tetracycline concentrations and mass spectrometry (MS) analyses indicated that the resultant transformation products primarily consisted of lower molecular weight metabolites (Delius et al., 2022). Several studies have documented the biotransformation and inactivation of tetracyclines via pure bacterial isolates. Recently, a novel bacterial strain *Alcaligenes* sp. T17 capable of degrading tetracycline was isolated from wetland sediment (Chen X. et al., 2022). The maximum degradation rate of tetracycline was 94.35% and the authors also identified five potential biodegradation products, while proposing a possible degradation pathway (degrouping, oxidation and ring-opening).

In a recent study, quinone oxidoreductase TjhO5 along with the NADH-dependent epimerase TjhD4 catalyze the conversion of an enone to a cycloalkane in the A-ring of unconventional tetracyclines, employing milligram-scale biocatalysis (Supplementary Table S1, row iv). These findings highlight an unusual post-modification of atypical tetracyclines and facilitate further engineering and biocatalysis options to enrich the structural diversities of tetracyclines (Nie et al., 2021). As shown in Figure 1, possible biocatalytic reactions of tetracycline would be reduction or alkylation of the amide group of ring A by means of oxidoreductases or transferases, respectively, oxidation of the tertiary amine of ring A, as well as halogenation of ring D by halogenating enzymes or alkylation/acylation of the phenolic OH of the ring D.

### 2.3 Erythromycin A

A typical example of a macrolide antibiotic is erythromycin A (Figure 1Aiii), which was introduced into clinical practice in 1952. Macrolides are considered the safest antibiotics for humans, mainly affecting Gram-positive cocci and intracellular pathogens such as *Mycoplasma, Chlamydia,* and *Legionella* (Vardanyan and Hruby, 2016). The macrolide framework comprises a versatile 14-membered core structure containing both chemically active and inert substituents and sites. In semi-synthesis, bacterial natural macrolides are employed as a foundation for making structural changes to peripheral substituents. A commonly used chemical catalyst for erythromycin A is 4-(dimethylamino)pyridine used in a variety of nucleophilic reactions that functionalize its free hydroxyl groups (Zhu et al., 2006; Zhu et al., 2007; Liu et al., 2011). However, there is no documented information in the literature about biocatalytic alterations following a similar approach.

Inspired by chemically made modifications of erythromycin A (Undheim, 2020), we can assume that some functional groups in its structure are good candidates for enzymatic reactions (Figure 1Aiii): the keto-group in position C-10 could be reduced by alcohol dehydrogenases to the corresponding alcohols or to an oxime or amine in the reductive amination by transaminases, amine dehydrogenases, or imine reductases (Hollmann et al., 2021). Some erythromycin A analogs were synthetically made to increase their stability and prevent cyclization in the acidic gastric environment (Cyphert et al., 2017). Esterification of free hydroxyl groups of the sugars using lipases could contribute to the stability of the molecule (Figure 1Aiii), and examples of chemically made derivatives in this manner are known in the literature (Omura et al., 1992). Also, the oxidation of the dimethyl amino group and subsequent coupling reactions could afford new derivatives of the native erythromycin A structure (Figure 1Aiii).

### 2.4 Actinomycin D

Actinomycin D, also known as dactinomycin, is a renowned antibiotic known for its dual properties of combating bacterial infections and suppressing tumor growth. Since its introduction into clinical use in 1954, it has established itself as one of the earliest anticancer drugs, marking the first instance where an antibiotic also demonstrated anticancer efficacy. Administered via intravenous delivery, it finds application in the treatment of various medical conditions, including gestational trophoblastic disease, Wilm’s tumor, rhabdomyosarcoma, Ewing’s sarcoma, and more (Vardanyan and Hruby, 2016). Actinomycin D, derived from *Streptomyces* spp., is the most significant member of the actinomycins, a group of closely related chromopeptide antibiotics. Its chemical structure consists of a heterocyclic chromophore along with two cyclic pentapeptide lactone rings (Figure 1Aiv). The heterocyclic segment, a phenoxazine derivative, includes a quinonimine component, imparting both the compound’s coloration and its capacity for intercalation (Avendaño and Menéndez, 2015). Some chemical reactions of actinomycin D and its fragments include metal complexation (Ivanova and Spiteller, 2012), elimination and transformation of some groups of the heterocyclic chromophore (Sehgal et al., 1985), but the systematization of most commonly used catalysts was not possible because of their structural divergence and reaction outcomes.

To reduce its cytotoxicity, analogs of actinomycin D have been produced by directed mutasynthesis, semi-synthesis and total synthesis (Mauger et al., 1991), but so far, no works related to biocatalytic derivatization of actinomycin D have been published. In these non-biocatalytic methods, the actinomycin D peptide moieties were mainly modified by replacement of amino acids in the pentapeptide rings (Mauger et al., 1991; Zhang et al., 2010), while modification of chromophoric phenoxazine ring mainly concerned introducing an amino group in position C-2 and a carbon atom in position C-7, but also modifications in positions C-4, C-6, and C-8 of the phenoxazone ring (Sengupta et al., 1975; Sinha et al., 1979; Loborg et al., 1995) (Figure 1Aiv). Methylated actinomycin D in position C-7 of the phenoxazone ring was isolated from the fermentation broth of *Streptomyces* sp. KLBMP 2541, but the enzymes responsible for this biotransformation were not further described or used (Chen et al., 2013). According to the number of derivatives obtained by semi- or chemical synthesis, this molecule has great potential to be transformed via biocatalysis (Sengupta et al., 1975; Sengupta et al., 1979; Sinha et al., 1979; Zhang et al., 2010).

### 2.5 Vancomycin

The first discovered glycopeptide antibiotic was vancomycin, identified from the fermentation of *Amycolatopsis orientalis* in 1956 (Figure 1Av). Vancomycin is categorized as a final option medication for addressing Gram-positive bacterial infections that have become resistant to other treatments. It is the antibiotic of first choice for treating infections caused by methicillin-resistant *Staphylococcus aureus* (MRSA) (Cong et al., 2020). To explore the SAR of vancomycin and find more effective new chemical entities, many different types of vancomycin derivatives were synthetically prepared and studied (Ma et al., 2020). A common chemical reagent reported for the transformation of vancomycin is diisopropylethylamine (DIPEA) used in click chemistry reactions (Silverman et al., 2017), alkylations (Okano et al., 2014; Wu et al., 2020), amide bond formation (Gong et al., 2006; Yarlagadda et al., 2014; Yarlagadda et al., 2016). Its complex structure includes several phenols susceptible to oxidation and eighteen asymmetric centers, thus requiring mild conditions for further derivatization (Marshall, 1965), so biocatalysis is considered a good choice for obtaining new derivatives.

**FIGURE 2:**
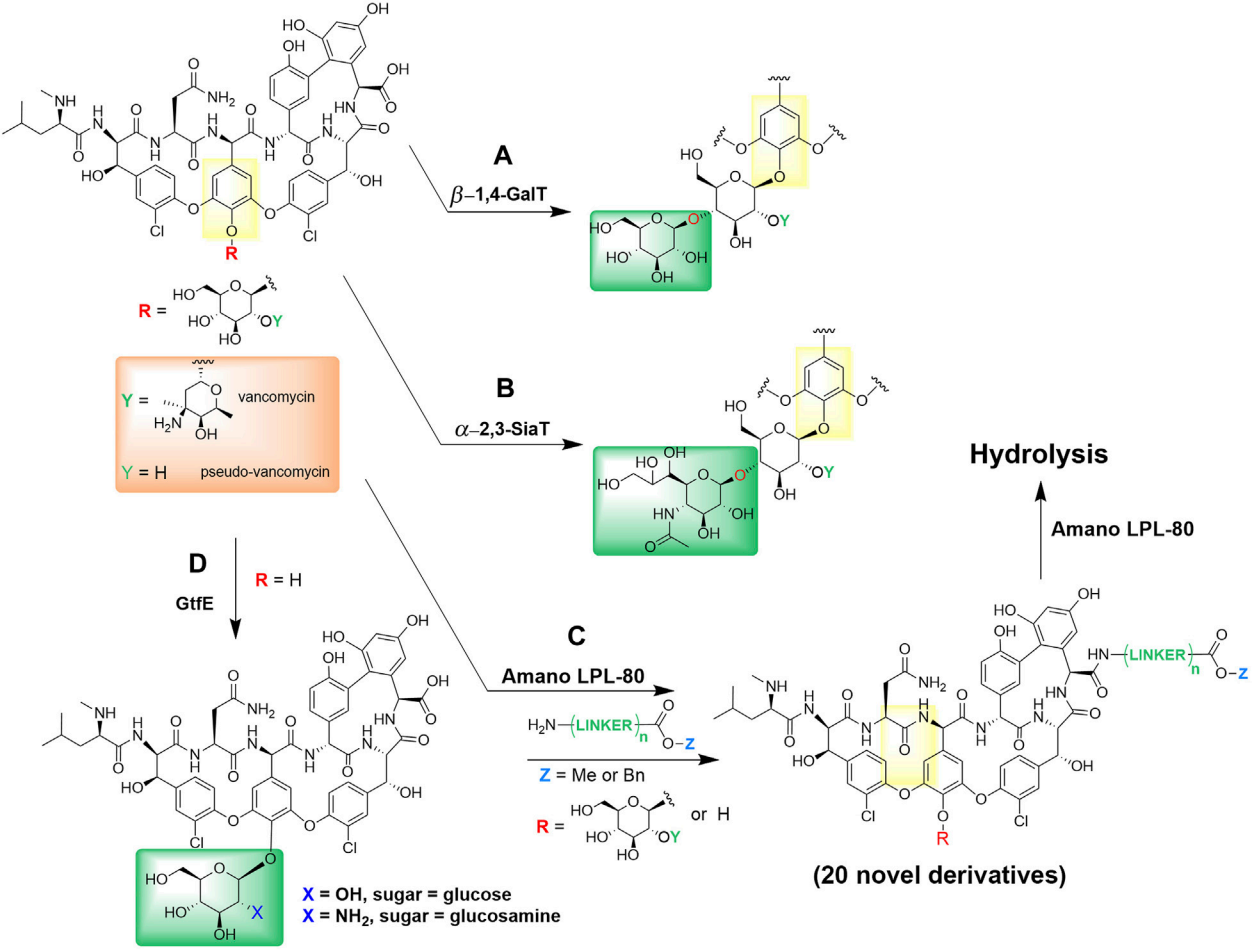
Enzymatic derivatization of vancomycin. **(A)** Enzymatic incorporations of galactose by *β*-1,4-galactosyltransferase and **(B)** sialic acid by *α*-2,3-sialyl transferase into vancomycin and pseudo-vancomycin (Oh et al., 2011). **(C)** Synthesis and hydrolysis of various vancomycin esters by Amano LPL-80 lipase from *Pseudomonas* sp. (Adamczyk et al., 1998; Thayer and Wong, 2006). **(D)** One-pot enzymatic glycosylation of vancomycin aglycon using glycosyltransferase (GtfE) (Thayer and Wong, 2006).

Recent enzymatic and chemo-enzymatic modifications have revealed that vancomycin’s disaccharide attachment may be one access point for an alteration route in pursue of new bioactive compounds (Losey et al., 2002). Analogs of the disaccharide vancomycin and the monosaccharide pseudo-vancomycin with new sugar attachments have been enzymatically synthesized by glycosylation with overexpressed glycosyltransferases, namely, *β*-1,4-galactosyltransferase (Figure 2A) and *α*-2,3-sialyl transferase (Figure 2B). All four derivatives, including galactose- and sialic acid-containing derivatives were described and characterized (Oh et al., 2011). In addition, antibiotic activities of galactose-containing derivatives against the MRSA and vancomycin susceptible *Enterococcus faecalis* were the same or better than those of sialic acid-containing derivatives. This enzymatic glycosylation strategy was performed on a milligram scale, and may also apply to other glycopeptides and other natural glycosylated small molecules, including polyketides and nonribosomal peptides.

Amano LPL-80 lipase from *Pseudomonas* sp. was used both for the esterification and hydrolysis of various vancomycin esters (Figure 2C), such as alkyl and benzyl esters without degradation (Adamczyk et al., 1998). Furthermore, Thayer and Wong reported a convenient chemo-enzymatic one-step method for producing vancomycin analogs containing glucose or *N*-alkyl glucosamine (Thayer and Wong 2006). These analogs demonstrated significant enhancements in antibiotic effectiveness against a strain of *Enterococcus* that had developed resistance to vancomycin. Their approach involved utilizing vancomycin aglycon as the base for glycosylation, during which they introduced native (glucose) and non-native (glucosamine) monosaccharides through the action of the glycosyltransferase enzyme (GtfE) onto the phenolic hydroxyl group (Figure 2D). This chemo-enzymatic method of natural products containing phenols may be useful for creating other glycosylated natural product analogs, *e.g.*, glycopeptides.

## 3 Antifungal compounds

During the last few decades, a large increase in systemic fungal infections has been observed as one of the key contributing factors of human mortality worldwide, with immunocompromised patients being the most susceptible group (Su et al., 2018). The three most common fungal pathogens, *Candida albicans*, *Cryptococcus neoformans*, and Aspergillus fumigatus, cause more than one million deaths in a year’s time, representing a significant threat to global health. The treatment of fungal infections using common antifungals, polyenes, azoles, peptidyl nucleoside antibiotics, echinocandins, allylamines, and flucytosine demonstrated drawbacks such as toxicity, limited efficacy, safety, and subpar pharmacokinetic properties (Su et al., 2018). In addition, long-term application and wide use of these antifungals led to a notable increase in drug resistance. Hence, it is paramount to innovate and create new antifungals for the successful treatment of fungal infections.

### 3.1 Amphotericin B

Amphotericin B is a broad-spectrum antifungal antibiotic with a polyene macrolide structure (Figure 1Bi), produced by *Streptomyces nodosus.* Despite being one of the oldest antifungals, with almost 5 decades of clinical use, the most widespread fungal pathogens have still not developed resistance as it is still administered only intravenously in a hospital setting (Cavassin et al., 2021). Amphotericin B binds to ergosterol, thereby disrupting fungal membranes by creating transmembrane pores that facilitate unregulated removal of small molecules and ions, leading to apoptosis (Mesa-Arango et al., 2012). Binding to cholesterol in mammalian membranes, as well as low solubility in water, lead to acute toxicity, which limits the full therapeutic utilization of amphotericin B (Murphy et al., 2010). In order to partially mitigate the toxicity, liposomal formulation or chemical structural modification can be used, but the high cost heavily restricts their utilization in undeveloped countries in the world (Carmody et al., 2005). Common chemicals for the transformation of amphotericin B are hydrochloric acid and sodium cyanoborohydride used for the alkylation of the primary amino group of the sugar moiety.

No reports of the biocatalytic reactions using amphotericin B have been reported to date, but using biocatalysis to modify the existing drug (Figure 1Bi), either via alkylation of the amino group of the sugar, or via oxidation or reduction of the carboxylic moiety, might be a way to reduce the negative effects, while simultaneously improving their activity.

### 3.2 Nystatin

Nystatin is an antifungal agent synthesized by strains of *Streptomyces noursei*, that has fungicidal and fungistatic properties and is utilized against a wide range of fungal and yeast infections (Khalandi et al., 2020). Its polyene macrolide structure strongly resembles that of amphotericin B, with minor differences (Figure 1Bii). It possesses superior antifungal properties, but its high toxicity still limits its use for the treatment of systemic infections (Rai et al., 2022). Nystatin also strongly binds to ergosterol in the plasma membrane of fungi, and forms pores that cause the leakage of essential cellular components, leading to the disturbance of electrochemical gradients, and ultimately resulting in cell death (dos Santos et al., 2017). Often used chemical for nystatin transformation is trimethylamine for the alkylation of the primary amino group of the sugar moiety.

Based on the literature search, there have not been any published reports regarding the biocatalysis of nystatin, but potential biotransformations may include decarboxylation and esterification of the carboxylic group located at position C-36 of the polyene structure. Also, oxidation at C-23 and C-24 via oxygenases might be possible to produce a more rigid and conjugated polyene like the one found in amphotericin B.

### 3.3 Natamycin

Natamycin is a tetraene polyene antifungal antibiotic produced by *Streptomyces natalensis* and *Streptomyces chattanoogensis* (Figure 1Biii) (Meena et al., 2021) and its structure contains a polyene macrocyclic lactone ring bonded to a mycosamine moiety (Figure 1Biii). It is used for the treatment of fungal infections in humans and is effective against nearly all yeast and fungi, including *Candida, Aspergillus, Cephalosporium, Fusarium and Penicillium* (Meena et al., 2021). Similar to other polyene antifungal compounds, it interacts with ergosterol in the plasma membrane. However, contrary to the majority of other polyene antifungals that change the permeability of cell membranes, natamycin mechanism of action includes stopping the ergosterol-dependent fusion of vacuoles (Te Welscher et al., 2010). A commonly used chemical for natamycin derivatization is trimethylamine used for the alkylation of the primary amino group of the sugar moiety.

Based on the literature search, there have not been any published reports regarding biocatalysis for natamycin, but potential biotransformations may include esterification of the carboxylic group located at position C-25 of the polyene structure, as well as alkylation/esterification of the primary amino group. A commonly used chemical for natamycin derivatization is trimethylamine used for the alkylation of the primary amino group of the sugar moiety.

### 3.4 Nikkomycin Z

Nikkomycins are a group of antifungal antibiotics, originally generated by *Streptomyces tendae* and *Streptomyces ansochromogenes*, that inhibit membrane-integrated chitin synthase (Chs), and stop the synthesis of chitin, a linear *N*-acetylglucosamine polymer that is a vital component of fungal cell walls (Liao et al., 2009; Wu et al., 2022). Nikkomycin Z (Figure 1Biv) has the highest activity and is made of 4-(4′-hydoxy-2′-pyridinyl)-homothreonine and a 5-aminohexuronic acid bonded to uracil via a *N*-glucosidic bond (Liao et al., 2009). Nikkomycin Z is a structural analog of UDP-*N*-acetylglucosamine, which is used by Chs in the production of chitin (Chen W. et al., 2022) and this structural similarity allows nikkomycin Z to act as a competitive inhibitor of the Chs. It has a synergistic effect when combined with additional antifungal compounds that affect the fungal cell wall, such as echinocandins which inhibit the FKS (membrane-integrated synthase) domain and the triazoles that inhibit the ergosterol biosynthesis (Hasim and Coleman, 2019). When chemical reactions are concerned, nikkomycin Z was only reported to undergo hydrolysis.

Decker et al. demonstrated the enzymatic bromination of the hydroxypyridyl group of nikkomycin Z, on a 5 mM scale, using a nonheme bromoperoxidase, isolated from *S. aureofaciens* Tü 24 (Supplementary Table S1, rows v and vi) (Decker et al., 1991). The mono- and dibrominated analogs showed decreased antifungal activity compared to nikkomycin Z, which might be a result of weaker interaction with the Chs enzyme. Although the enzyme mechanism of action was not yet elucidated, *in vitro* bromination of nikkomycin Z using a nonheme bromoperoxidase would not occur without the presence of the acetate anion. No further biocatalytic reactions were performed on this molecule, but carboxylic group reduction or derivatization of the primary amino group could be envisaged.

### 3.5 Pyrrolnitrin

Pyrrolnitrin (Figure 1Bv) is a BNP with a unique structure that contains both a pyrrole ring and a nitro functional group, and a has broad-spectrum of antibiotic and antifungal properties (Pawar et al., 2019). It was first isolated in 1948 from *Pseudomonas pyrrocinia*, and later from a variety of *Pseudomonas* spp. and *Burkholderia cepacia* (Jung et al., 2018; Pawar et al., 2019). Pyrrolnitrin has been found to have plant growth-promoting properties, including increasing root growth and inducing systemic resistance to pathogenic fungi in plants (El-Saadony et al., 2022). A few chemical reactions of pyrrolnitrin are known and mostly include functionalization of the nitrogen of the pyrrole ring (Koyama et al., 1987; Sako et al., 2002), as well as dehalogenation (Sako et al., 2001), but the systematization of most commonly used catalysts was not possible, as they are few and differ much in structure.

Bongs and van Pée managed to perform chlorination of pyrrolnitrin at 50 µM scale by utilizing substrate-specific non-heme haloperoxidases from *P. pyrrocinia* and *S. aureofaciens* Tü24 (Bongs and van Pée, 1994). The major product of the chlorination was 2-chloropyrrolnitrin, while the structure of the second product was proposed to be 2,5-dichloropyrrolnitrin, as its structure could not be determined by ^1^H-NMR (Supplementary Table S1, row vii). The latter structure was proposed according to the postulated enzyme mechanism of action, which includes the electrophilic aromatic substitution by haloperoxidase-bound Cl^+^, making it more probable that the chlorination would occur on the pyrrole ring of pyrrolnitrin, and not the benzene ring that is strongly deactivated by the nitro group.

## 4 Anticancer compounds

According to the WHO (World Health Organisation), cancer is a leading cause of death worldwide, accounting for nearly one in six deaths in 2020 (https://www.who.int/news-room/fact-sheets/detail/cancer). The commonly used approaches in cancer treatment include surgery, radiotherapy and chemotherapy using approved drugs, where anticancer drugs are classed as cytotoxic since they also affect healthy cells and cause a series of side effects (Patrick, 2013). Based on their mechanism of action, anticancer drugs are separated into several categories: drugs acting directly on nucleic acids (*e.g*., doxorubicin, also called adriamycin, and daunorubicin, also called daunomycin or rubidomycin), drugs acting on enzymes involved in DNA synthesis (antimetabolites), hormone-based therapies, drugs acting on structural proteins, inhibitors of signaling pathways, miscellaneous enzyme inhibitors and miscellaneous anticancer agents. (Patrick, 2013).

### 4.1 Doxorubicin

Doxorubicin (Figure 1Ci) belongs to a group of naturally occurring antibiotics anthracyclines and is one of the most effective anticancer drugs ever discovered. It was first isolated from *Streptomyces peucetius* in 1967 which was discovered in a soil sample taken from the shore of the Adriatic Sea (thus the alternative name adriamycin), it contains a tetracyclic system where three of the four rings are planar and it entered the clinical practice soon after its discovery (Patrick, 2013). Most commonly used catalyst for chemical reactions of doxorubicin is the trifluoroacetic acid and the reaction takes place exclusively at the carbonyl moiety (Figure 1Ci), followed by another synthetic catalyst trimethylamine, where the reaction can take place either at the carbonyl group, or the amino group, or on the substituents of the planar portion of the molecule.

**FIGURE 3:**
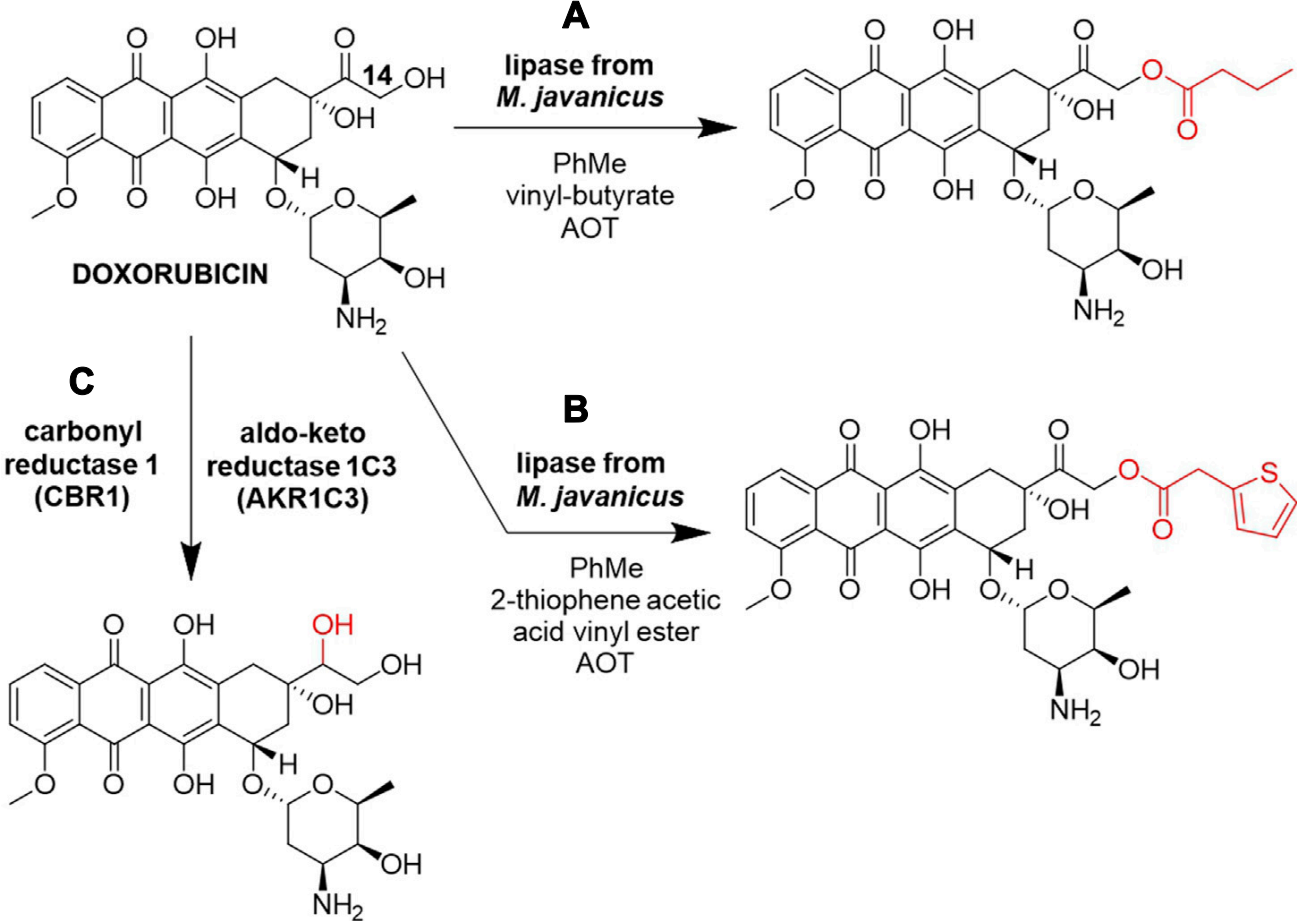
Biocatalytic reactions of Doxorubicin. **(A)** Acylation by lipase from *M. javanicus* using vinyl butyrate (Altreuter et al., 2002). **(B)** Acylation by lipase from *M. javanicus* using 2-thiophene acetic acid vinyl ester (Altreuter et al., 2002), **(C)** carbonyl reduction by carbonyl reductase 1 (CBR1) and aldo-keto reductase 1C3 (AKR1C3) (Piska et al., 2021).

The primary hydroxyl (C14-OH) of doxorubicin was successfully acylated with vinyl butyrate or 2-thiophene acetic acid vinyl ester by lipase from *Mucor javanicus* (Figures 3A, B, respectively) (Altreuter et al., 2002). These site-specific acylations were performed in a non-aqueous solvent toluene using the anionic surfactant Aerosol OT (AOT, docusate sodium) to enhance solubilization and activate the commercial lipase. The reaction was performed on a 0.20 mg (0.37 µmol) doxorubicin scale and product detection was done by LC-MS. This work expanded the potential use of non-aqueous enzymology in drug discovery.

Many anthracycline resistance mechanisms have been revealed, commonly including their biotransformation to less active metabolites (Piska et al., 2019). Carbonyl reduction *in vitro* of various anthracyclines at 20 µM scale, including doxorubicin, in the human liver cytosol by carbonyl reductase 1 (CBR1) and aldo-keto reductase 1C3 (AKR1C3) was described as a mechanism of intrinsic clearance of anthracyclines (Figure 3C). The existence of an additional side-chain hydroxyl group next to the carbonyl moiety undergoing reduction was recognized as the major factor in defining anthracyclines susceptibility to carbonyl reduction, slowing down the metabolism of anthracyclines that contain this vicinal hydroxyl group, such as doxorubicin and epirubicin, which was observed by UPLC-MS/MS (Piska et al., 2021). Despite these reduced metabolites being deemed undesirable for cancer treatment (Piska et al., 2019), this approach offers a fresh point of view in the study of late-stage anthracyclines biotransformation that could find use in the discovery of novel anthracycline analogs. Furthermore, the carbonyl group of doxorubicin is a potential spot for reductive amination by transaminases, which could alter the metabolic stability of this drug, but further biocatalytic alkylations/acylations could happen on the hydroxyl and the amino groups of the sugar moiety (Figure 1Ci).

Aiming at the removal of doxorubicin from the environment, a successful biotransformation was reported for the white-rot fungus *Bjerkandera adusta* CCBAS 930 (whole cells) by means of native peroxidases: horseradish-type peroxidase, manganese-dependent peroxidase, lignin peroxidase and versatile peroxidase. More than 90% of doxorubicin was removed by biodegradation and this biotransformation monitored through enzymatic decolorization gave non-colored oxidation products, including some phenolic compounds and free radicals. Although being an example of doxorubicin biocatalysis this biotransformation was designed as a bioremoval and detoxification protocol and the formed products were not used further (Rybczyńska-Tkaczyk et al., 2021). The same group designed immobilized mycelium cultures of *B. adusta* CCBAS 930 containing similar versatile peroxidase (VP), superoxide dismutase (SOD), catalase (CAT) and glucose oxidase (GOD) for the removal of doxorubicin from the environment. The formed degradation products included phenolic compounds (hydroxyphenols), antioxidants and free radicals, where again *c. a.* 90% of doxorubicin was removed after 120 h and the immobilized cells kept their degradation potential for up to 12 weeks (Rybczyńska-Tkaczyk, 2021).

Another study of the *in vitro* degradation of doxorubicin using a postmitochondrial fraction (PMF) of homogenized Fisher 344 rat liver tissue containing microsomes and soluble cell components, including cytochrome P450 enzymes, carbonyl reductase, NADPH-P450 reductase and NAD(P)H/quinone oxidoreductase, combined HPLC with a tandem laser-induced fluorescence (LIF) and MS detection and identified different metabolites of DOX, including 7-deoxydoxorubicin aglycone (Supplementary Table S1, row viii) and 7-deoxydoxorubicinol aglycone (Supplementary Table S1, row ix) (Katzenmeyer et al., 2010). When biocatalytic systems were cytosolic fractions containing dominantly carbonyl reductases, doxorubicinol was detected as the major metabolite (Piska et al., 2019; Piska et al., 2021), but in the case of the PMF fraction that includes microsomes, doxorubicin and doxorubicinol are rapidly converted by NADPH-P450 reductase and NAD(P)H/quinone oxidoreductase to aglycone metabolites with the cleaved daunosamine sugar (Supplementary Table S1, rows viii and ix), which offers a different metabolic profile of this PMF biocatalytic system.

### 4.2 Daunorubicin

Daunorubicin (Figure 1Cii) is a structure very similar to doxorubicin and was also first isolated from *S*. *peucetius* in the 1960s, diverse only in one vicinal hydroxyl group (Patrick, 2013). Commonly used chemical catalysts for daunorubicin transformations are 4-(dimethylamino)pyridine and *N*, *N*′-diisopropylcarbodiimide, mostly used for reactions on the primary amino group of the sugar moiety.

Aforementioned *in vitro* carbonyl reduction of various anthracyclines, including doxorubicin (Figure 3C), in the human liver cytosol by CBR1 and AKR1C3 indicated that the absence of the vicinal hydroxyl group advanced biocatalytic reduction of anthracyclines, which is why daunorubicin (Supplementary Table S1, row x) was metabolized faster than doxorubicin by these enzymes (Piska et al., 2021). Carbonyl reduction of daunorubicin to less cytostatic but more cardiotoxic daunorubicinol has been associated with cardiotoxicity and drug resistance, in particular with the pharmacokinetic efflux mechanism from cancer cells through ATP-binding cassette transporters. The carbonyl reducing enzyme belonging to aldo-keto reductase (AKR) superfamily, AKR1C3 was assessed as a target for two cyclin-dependent kinase inhibitors AZD5438 and R547, and it was shown that they both prevented daunorubicin biotransformation to daunorubicinol, thus positively affecting its efficacy and safety (Sorf et al., 2019).

The same biotransformation product (daunorubicinol) was observed when the reduction of various xenobiotics containing a carbonyl group was assessed using homogenate of nematode *Haemonchus contortus* (cytosol-like subcellular fractions), one of the most pathogenic parasites of sheep and goat (Cvilink et al., 2008). Aforementioned immobilized mycelium cultures of *B. adusta* CCBAS 930 containing VP, SOD, CAT and GOD were also used for the bioremoval daunorubicin from the environment and degradation products included hydroxyphenols, antioxidants and free radicals (with no particular structural characterization), where *c. a.* 86% of daunorubicin was removed after 120 h, and the detailed characterization of this VP could provide valuable information when removal of harmful xenobiotics from the environment is concerned (Rybczyńska-Tkaczyk, 2021).

In addition to approved and clinically used drugs such as doxorubicin and daunorubicin, a vast number of anticancer compounds, including some BNPs, are in pre-clinical trials, and the purpose for their modification would be improving the potential for their medical use, especially in the field of anticancer therapy (Guo, 2017).

### 4.3 Prodigiosin

Prodiginines are a class of tripyrrolic compounds produced by the secondary metabolism of various Gram-positive (*Streptomyces* spp.) and Gram-negative (*Serratia* and *Vibrio* spp.) bacteria, that have been attracting attention due to their prevalently anticancer activity, but also numerous others (Williamson et al., 2007). Prodiginines have either linear substituents, like prodigiosin and undecylprodigiosin (Figure 1Ciii), or cyclic ones, like cycloprodigiosin (methyl-*ortho*-cyclohexyl-prodigiosin), streptorubin B (butyl-*meta*-cycloheptyl-prodigiosin) or streptorubin A (ethyl-*meta*-cyclononyl-prodigiosin) (Stankovic et al., 2014; Klein et al., 2018). Their application is hindered by low selectivity between healthy and cancer cells, so structural optimization to increase selectivity indices is needed. In our previous work, two novel brominated derivatives were obtained via late-stage modification by applying mild green reagents such as hydrogen peroxide and 48% hydrobromic acid in methanol (Lazic et al., 2022), while other works studied prodigiosin mainly as a ligand for metal complexation.

Prodigiosin has been researched for decades and its numerous biological activities have been reported (Lin et al., 2020), as this biopigment can be produced by many bacterial strains by means of biotechnology (Han et al., 2021). Although the electron-rich nature of prodigiosin is evident (Figure 1Ciii) and its UV-protective properties have already been studied (Borić et al., 2011), electrophilic enzymatic reactions, such as halogenation, nitration, alkylation, acylation are not mentioned in the literature. Nevertheless, the existence of cycloprodigiosin suggests that successful C–H activation and oxidative cyclization of the side chain of prodigiosin (Figure 1Ciii) is possible by means of hydroxylase enzymes (de Rond et al., 2017), although these enzymes were not utilized as biocatalysts on a larger scale. This type of oxidative cyclisation is not easily achieved under standard organic synthesis reaction conditions and would make this type of enzyme a much appreciated addition to the plethora of oxidative biocatalysts, which predominantly consist of P450 enzymes, but includes peroxygenases, monooxygenases and hydroxylases (Romero et al., 2021).

### 4.4 Undecylprodigiosin

No chemical reactions of undecylprodigiosin are known to date and no biocatalysis was reported for this molecule. Analogous to the cyclization of prodigiosin to cycloprodigiosin, enzymes that catalyze the cyclization of undecylprodigiosin from *Streptomyces* spp. to streptorubin B or streptorubin A (Figure 1Civ) have been described as Rieske nonheme iron-dependent oxygenase-like enzymes (Sydor et al., 2011). They are considered participants in the degradation of aromatic compounds (Sydor and Challis, 2012), so this oxidative cyclization principle is an enzymatic reaction which could be utilized in biocatalysis or on other BNPs containing long alkyl chains, thus affording semi-synthetic natural product derivatives. In addition to prodigiosin (Figure 1Ciii) and undeclyprodigiosin (Figure 1Civ), several other prodiginines, such as streptorubin A and B, cycloprodigiosin and roseophilin are known BNPs, but like for undecylprodigiosin - no further biocatalytic reactions were reported for these molecules.

### 4.5 Staurosporine

Staurosporine (Figure 1Cv) is a secondary metabolite that revolutionized anti-cancer therapy as a potent, but non-specific inhibitor of tyrosine kinases. It was discovered in a soil-dwelling microbe *Streptomyces staurosporeus* (now *Lentzea albida*), and its indolocarbazole structure was proposed in 1978, but confirmed in 1994 (Ōmura et al., 2018). A commonly used chemical catalyst for staurosporine transformation is the chemical 4-(dimethylamino)pyridine and in the majority of cases, the reaction takes place at the amino group of the sugar, or less often at the nitrogen which is part of the lactam ring.

Staurosporine was not utilized in late-stage biocatalysis, but it could be used in enzymatic alkylation/acylation reactions of its secondary amine groups, halogenation reactions by means of halogenases or haloperoxidases, or oxidation (Figure 1Cv).

## 5 Immunosuppressive compounds

Immunosuppressive drugs hinder abnormal immune responses by suppressing immune cell proliferation and function and are predominantly utilized in organ transplantation as anti-rejection medicaments and for autoimmune diseases. Numerous signaling pathways have been identified as pivotal in orchestrating the cascade immune response and various targets have been identified for the development of immunosuppressive agents, but despite the notable treatment success, there is continuous research and development potential in this area (Xu and Chu, 2022).

### 5.1 Rapamycin

Rapamycin (sirolimus, Figure 1Di) is a BNP that possesses powerful immunosuppressive properties and is produced by *Streptomyces hygroscopicus* (Abraham and Wiederrecht, 1996). Rapamycin is currently used in immunosuppressant therapy following organ transplantation (Graziani, 2009). In addition, its analogs (rapalogs) are either in clinical use (everolimus and temsirolimus) or undergoing clinical trials (ridaforolimus and DL001) for the treatment of neurodegenerative diseases and cancer treatment (Kuerec and Maier, 2023). The abovementioned semisynthetic rapalogs everolimus and temsirolimus (42-ester rapamycin derivatives) were developed to improve the pharmacological properties of rapamycin and have been approved in the treatment of several different types of carcinoma (Kuerec and Maier, 2023). Total syntheses of rapamycin and rapalogs display little efficiency and are not considered effective in the development of novel therapeutic agents. In contrast, there has been considerable interest in the regioselective modification of rapamycin using precursor directed biosynthesis (Graziani et al., 2003), mutasyntesis (Gregory et al., 2005), as well as late-stage biocatalytic methods (Gu et al., 2005; Gu et al., 2005). Most of the chemical reactions of rapamycin were preformed using 4-(dimethylamino) pyridine as the catalyst and referred to functionalization of the hydroxyl groups in C-42 and/or C-28 positions.

**FIGURE 4:**
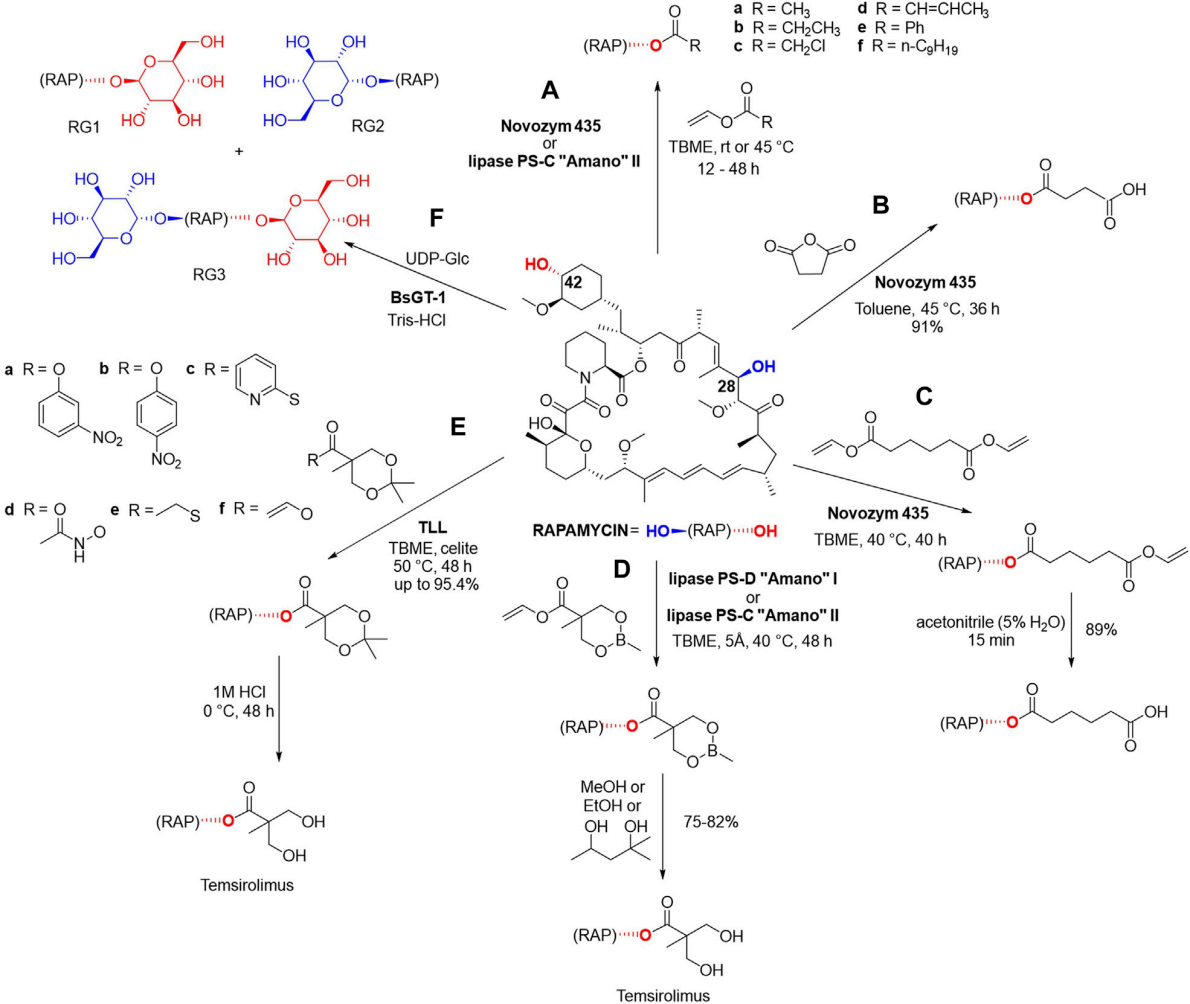
Enzymatic esterification of rapamycin at 42-hydroxyl position in the presence of one of the following lipases: Novozym 435, lipase PS-C “Amano” II, lipase PS-D “Amano” I and **(A)** vinyl acetate, vinyl propionate, vinyl 1-chloroacetate, vinyl crotonate, vinyl benzoate and vinyl decanoate (Gu et al., 2005) **(B)** succinic anhydride in toluene (Gu et al., 2005), **(C)** divinyl adipate in TBME in the first step and acetonitrile in the second step (Gu et al., 2005). Biocatalytic synthesis of temsirolimus using **(D)** cyclic methylborate protected vinyl ester in the first step, and alcoholic solvents such as MeOH, EtOH, 2-methylpentane-2 or -5-diol for the deprotection in the second step (Gu et al., 2005), **(E)** ketal-protected vinyl esters in the first step, and a subsequent acid-catalyzed deprotection (Ju et al., 2015). **(F)** Glycosylation of rapamycin using glycosyltransferases BsGT-1 and UDP-Glc as the sugar donor with three possible products (Zhang et al., 2020). These reactions yielded 18 novel rapamycin derivatives.

Gu et al. have demonstrated that rapamycin can be regioselectively acylated at 42-hydroxyl position using either lipase B from *Candida antarctica* (Novozym 435) or the immobilized enzyme lipase PS-C “Amano” II from *B*. *cepacia* in anhydrous *tert-*butyl methyl ether (Figure 4A). The reaction with vinyl acetate, vinyl propionate and vinyl 1-chloroacetate were completed within 12–48 h at room temperature or at 45 °C with less reactive vinyl crotonate, vinyl benzoate and vinyl decanoate (Figure 4A) (Gu et al., 2005).

The reaction of rapamycin with succinic anhydride in toluene was carried out using 2 g of the starting material, the corresponding 42-hemisuccinate was obtained in a yield of 91% after 36 h and its structure was confirmed via Fourier transformation-ionic resonance mass spectrometry (FT-ICRMS) as well as ^1^H and ^13^C NMR (Figure 4B). The enzymatic synthesis of rapamycin 42-hemisuccinate using Novozym 435 had been reported in multiple analytical scale studies as a starting point for obtaining bioconjugates. 42-Hemisuccinate was further modified using synthetic reagents such as *N*-hydroxysuccinimide which gave rise to an activated ester that can be further conjugated to protein carriers (Behrouz et al., 2016), therapeutics via linker molecules (Zhang et al., 2019) or gelation agents to form precursors of hydrogelators (Li et al., 2012).

Novozym 435 was successfully used in another chemo-enzymatic reaction using divinyl adipate, followed by hydrolysis, and 89% yield of 42-hemiadipate was achieved (Figure 4C). This biocatalytic reaction was also carried out on a Gram scale, using 3 g of rapamycin and the structure of the derivate was confirmed via FT-ICRMS, ^1^H and ^13^C NMR (Gu et al., 2005).

Three chemo-enzymatic approaches were reported for the synthesis of the previously mentioned antitumor agent temsirolimus using immobilized lipases (Figures 4D–F). The first procedure was conducted utilizing 3 g of rapamycin and made use of a cyclic methylborate as the protecting group, and alcoholic solvents such as MeOH, EtOH, 2-methylpentane-2,5-diol for its deprotection, with yields of 75%–82% (Figure 4D).

In another chemo-enzymatic strategy for obtaining temsirolimus (Figure 4E), 1 g of rapamycin was reacted with ketal-protected vinyl esters as acyl donors using *Thermomyces lanuginose* lipase (Immozyme Tll) in TBME for 48 h at 50°C (Ju et al., 2015). Through the optimization of the reaction conditions, including temperature, solvent and additives, the reaction of 6 acyl donors was optimized to achieve yields up to 95.4%. The product was subsequently subjected to acid-catalyzed deprotection using 1 M HCl to yield temsirolimus. This study showcases a cost-effective, efficient, and scalable approach for the synthesis of temsirolimus.

In an attempt to improve rapamycin’s water solubility and bioavailability Zhang et al. reported a series of enzymatically catalyzed glycosylation reactions using glycosyltransferases from *B. subtilis* (BsGTs, Figure 4F) preformed on a small-scale analytical setup (Zhang et al., 2020). BsGT-1 was the most effective biocatalyst and UDP-D-glucose (UDP-Glc) was the only sugar donor accepted by this enzyme (Zhang et al., 2020). Rapamycin was dissolved in DMSO (0.2 mM final concentration) and the reactions were performed in 50 mM Tris-HCl buffer, 10 mM MgCl_2_ in the presence of 1 mM UDP-Glc and 500 μg/mL of the enzyme. Glycosylation occurs at C-42 yielding rapamycin-42-*O*-β-D-glucoside (RG1), at C-28 yielding rapamycin-28-*O*-β-D-glucoside (RG2), or in both these positions affording rapamycin-28,42-*O*-β-D-diglucoside (RG3), which were identified using Ultra performance liquid chromatography photodiode array (UPLC-PDA) detection at 278 nm and NMR spectrometry. Glycosylation products displayed low cytotoxicity, but have lost their antifungal, antitumor, and immunosuppression bioactivities, especially in the case of the C-28 glycosylation products RG2 and RG3 (Zhang et al., 2020). Most of the chemical reactions of rapamycin were preformed using 4-(dimethylamino) pyridine as the catalyst and referred to functionalization of the hydroxyl groups in C-42 and/or C-28 positions.

### 5.2 FK506

FK506 (tacrolimus, Figure 1Dii) is an FDA-approved immunosuppressive drug originally produced by *Streptomyces tsukubensis*. It is currently used in the treatment of patients undergoing organ transplants, but also displays the potential to be used as an antifungal, neuroprotective and neuroregenerative agent (Jung et al., 2021), and as therapy for conjunctivitis (Na et al., 2016). However, in order to broaden its use to neuronal diseases, conjunctivitis and as an antifungal, its immunosuppressive activity must be abolished and its water solubility improved. Many chemical reactions of FK506 are enabled by transition metal catalysts on the basis of Ru(II) (Grubbs’ catalyst) used mainly for olefin metathesis reactions (Clemons et al., 2002; Marinec et al., 2009), or Pd(II) which can afford various coupling reaction derivatives (Wang et al., 2019).

To address the issue of low water solubility, a two-step chemo-enzymatic process for the preparation of a water-soluble polyethylene glycol (PEG) conjugate of FK506 on an analytical scale (10 mg) was reported (Supplementary Table S1, row xi) (Gu et al., 2009; González-Sabín et al., 2011). In the first step, a functionalized α-bromoester is attached to the 32-hydroxyl moiety through a regioselective lipase-catalyzed reaction (González-Sabín et al., 2011). Then, the bromoacetate is treated with a thiol-terminated polyethylene glycol derivative under alkaline conditions affording the final product with a 83% yield (González-Sabín et al., 2011). The same process could be applied to obtain the corresponding C-42 PEG-analog of rapamycin (González-Sabín et al., 2011). The structure of the abovementioned derivatives was confirmed using ESI-MS. Na et al. have also reported obtaining two water-soluble conjugates of FK506 via a spontaneous reaction of FK506 with a succinyl linker followed by an enzymatically catalyzed conjugation of glucose (affording FK506-G, Supplementary Table S1, row xii) or amino carbohydrate sialic acid (FK506-S, Supplementary Table S1, row xiii) by glucosyltransferase or sialyltransferase, respectively, and these conjugates were quantified by HPLC analysis (Na et al., 2016).

## 6 *Quorum* sensing (QS) modulators

Parasitic microorganisms, particularly exemplified by *Pseudomonas aeruginosa,* deploy adaptive strategies, such as the *quorum* sensing (QS) controlled formation of biofilms and the production of countless, structurally diverse virulence factors (Díaz-Pérez et al., 2022). This enables the bacteria to function in a synchronized fashion (Lazar et al., 2021) which allows them to readily adapt to environmental changes and protect the biofilm integrity. Consequently, biofilm infections such as chronic airway infections in cystic fibrosis patients, are very difficult to eradicate and often require aggressive treatment (Papenfort and Bassler, 2016). Therefore, QS modulators are considered a valuable target for the treatment and prevention of bacterial infections, and their biotransformation could find a purpose in clinical practice.

### 6.1 Pyochelin

Pyochelin (Figure 1Ei) is a QS siderophore with a crucial function in microbial interactions and the overall fitness of *P. aeruginosa* spp. The inactivation of pyochelin production leads to decreased growth of *P. aeruginosa* spp. (Jenul et al., 2023). Few chemical reactions of pyochelin are known, mainly methyl esterification using diazomethanes (Rinehart et al., 1995; Ino and Murabayashi, 2001).

Esterification of the carboxylic acid moiety of pyochelin with glycolic acid (GA) was achieved on an analytical scale using a plant pathogen *Phellinus noxious* (Supplementary Table S1, row xiv) and it was suggested this esterification occurs in mixed cultures as a type of resistance response (Ho et al., 2021). This enzymatic modification leads to a decreased affinity of pyochelin-GA towards FtpA (ferripyochelin) receptors and a weaker iron binding, thus disabling iron acquisition by *P. aeruginosa* (Jenul et al., 2023).

Inactivation of pyochelin by Spm (staphylococcal pyochelin methyltransferase) was also observed when microbial interaction between *P. aeruginosa* and *S. aureus* was studied (Supplementary Table S1, row xv) (Jenul et al., 2023) and like for pyochelin-GA, pyochelin methyl ester had lower iron binding (Ho et al., 2021; Jenul et al., 2023). Both pyochelin-GA and pyochelin methyl ester were obtained on an analytical scale and identified using LC-MS techniques. In addition to esterase and transferase enzymes, the presence of carboxylic and phenolic groups in pyochelin suggests that hydrolases, such as lipases, could be used to facilitate the esterification of either of the groups (Figure 1Ei). Additionally, the phenolic group of pyochelin may also participate in an enzymatically catalyzed alkylation reaction, while the carboxylic group could undergo biocatalytic reduction (Figure 1Ei), although no such reports have been made to date.

### 6.2 Pyocyanin

Pyocyanin (Figure 1Eii) is involved in the QS system, it provides protection and ensures a competitive advantage to the bacteria which secrete it (Das and Manefield, 2012). It has feasible applications in medicine, pharmacy, as a biocontrol agent (Marrez and Mohamad, 2020; Saleem et al., 2021), as a redox indicator, in pH sensors, in the construction of organic light-emitting diodes (OLED) (Díaz-Pérez et al., 2022) and more, so the late-stage diversification could obtain novel derivatives with enhanced therapeutic or functional properties. Few chemical reactions of pyocyanin are known and include halogenation (Kohatsu et al., 2020) and conjugation reactions (Cheluvappa et al., 2008), but the systematization of most commonly used catalysts was not possible, as they differ much in structure.

There have been some reports of pyocyanin biotransformation, but not in the context of enzymatic structural optimization. The first report of pyocyanin reduction was observed only by amperometric detection in 1983, when glucose oxidase from *Penicillium vitale* was used (reaction not shown) (Kulys and Čénas, 1983). Since then, there have been reports of biocatalytic transformations using other oxidoreductases and hydrolase, which studied pyocyanin inactivation as a potential therapeutic approach.

It was reported that in the presence of microperoxidase 11 (MP11, heme peptide derived from cytochrome C) pyocyanin undergoes oxidation by hydrogen peroxide in just 30 min (Reszka et al., 2004). In this analytical scale reaction, hydrogen peroxide oxidizes MP11 to an analog compound containing an oxo-ferryl moiety and a π-radical cation, as reactive centers. Pyocyanin is irreversibly oxidized by the reactive centers of MP11 and it reduces the biocatalyst back to the ferric state via two one-electron transfer steps (Supplementary Table S1, row xvi). It was also shown that reducing compounds such as ascorbate could inhibit the MP11 enzyme and prevent the described oxidation process of pyocyanin. The reaction generates a pyocyanin radical formed by one-electron oxidation, which can then react to form dimers or give rise to alternative stable products of degradation.

An analytical-scale setup for generating mono-nitrated pyocyanin derivatives with altered biological activity has also been reported in the literature (Reszka et al., 2012). Pyocyanin was exposed to lactoperoxidase or myeloperoxidase and in the presence of hydrogen peroxide and sodium nitrite and the nitration yielded three green mononitrated analogs (Supplementary Table S1, row xvii) with the nitro-group in either positions C-2, C-4 or C-6 of the phenazine ring, with a maximum reaction rate measured at pH 6.4 (Reszka et al., 2012). The fraction containing all nitrated pyocyanin derivatives was assessed for its biological activity, and the results suggested that all nitrated derivatives have negligible antimicrobial activity and were devoid of their ability to cause inflammation in contrast to pyocyanin. In the same study, the effects of different ions were examined and it was shown that the same reaction took a significantly different turn in the presence of chloride. Using myeloperoxidase, monochlorinated isomers of pyocyanin, dichloropyocyanin, small amounts of two dibromoisomers of pyocyanin and a mixed bromochloropyocyanin species were obtained, all of which were detected by tandem MS, but the exact positions of the functional groups was not specified (Table X, row xviii) (Reszka et al., 2012).

The synthesis of both nitroaromatics and halogenated natural products has fundamental importance in pharmaceutical industries as these functional groups often play important roles in the action of certain drugs (Strauss, 1979; Jiang et al., 2016). Halogenation strategies involving the use of naturally occurring halogenases or haloperoxidases could be an effective route for generating novel bioactive analogues of pyocyanin, but there have been no reports regarding the use of these enzymes for the regioselective halogenation of pyocyanin.

Pyocyanin is also involved in biofilm formation and expansion and its selective biocatalytic degradation may facilitate treatment of infections (Costa et al., 2017). Costa et al. have described a previously unknown tautomerizing demethylase from *Mycobacterium fortuitum* (PodA) which can transform pyocyanin to 1-hydroxyphenazine and formaldehyde on an analytical scale, where the products were determined by HPLC (Supplementary Table S1, row xix). A novel reaction mechanism was proposed, where the change in the oxidation state of pyocyanin enables catalysis. This mechanism has not been perceived for demethylases before and suggests this enzyme utilizes the oxidized substrate as an electron acceptor. The kinetic analysis suggested a high affinity and specificity of the PodA enzyme over a wide array of pH values (4.9–8.7) and ionic strength (0–400 mM NaCl) (Costa et al., 2017).

### 6.3 1-Hydroxyphenazine

1-Hydroxyphenazine (Figure 1Eiii) could be used as the basis for developing novel bioactive molecules, as it possesses antibacterial, antifungal and anticancer activities (Wan et al., 2022). It is well known for its inhibitory effects on plant pathogens and has been recognized as a developing biocontrol agent (Wan et al., 2022). Chemical bromination of 1-hydroxyphenazine is successfully done using N-bromosuccinimide, and chemical alkylation/acylation of the OH group is performed using potassium carbonate.

**FIGURE 5:**
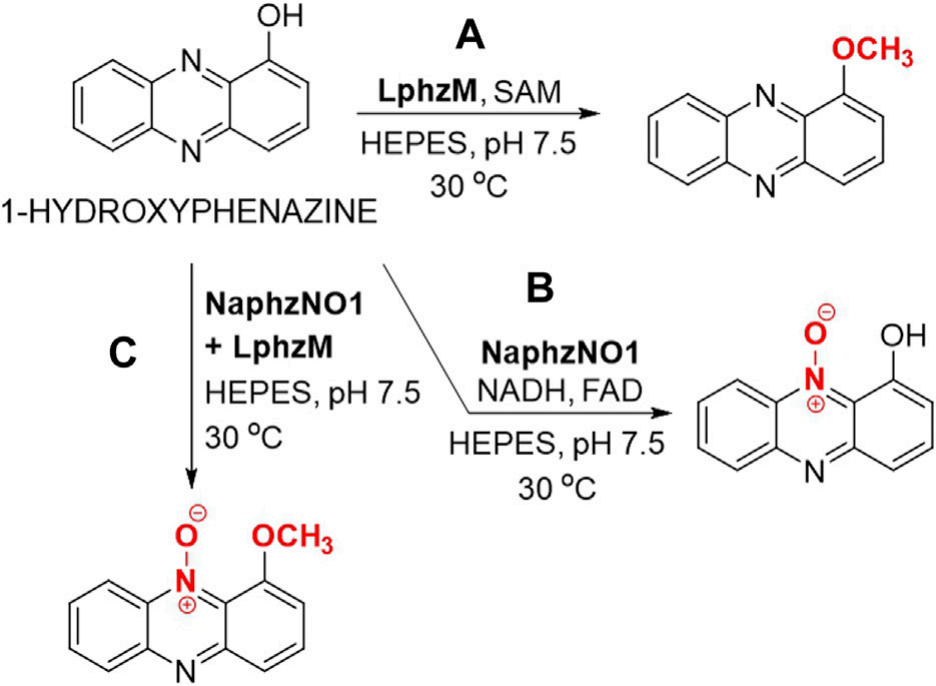
Enzymatic modification of 1-hydroxyphenazine through the use of a monooxygenase and/or methyltransferase. **(A)** Enzymatic methylation of 1-hydroxyphenazine using methyltransferase LphzM and *S*-adenosyl methionine (SAM) in HEPES (*N*-(2-hydroxyethyl)-1-piperazine-*N*’-(2-ethanesulfonic acid) buffer. **(B)** Enzymatic oxidation of 1-hydroxyphenazine using *N*-monooxygenase NaphzNO1 in the presence of nicotinamide adenine dinucleotide (NADH) and flavin adenine dinucleotide (FAD). **(C)** Enzymatic oxidation of 1-hydroxyphenazine using *N*-monooxygenase NaphzNO1 followed by methylation using methyltransferase LphzM in HEPES buffer (Wan et al., 2022).

Wan et al. reported the use of 1-hydroxyphenazine in reactions on an analytical scale using a recombinant methyltransferase from *Lysobacter antibioticus* and/or *N*-monooxigenase from *Nacardiopsis* sp. that have been expressed in *Escherichia coli* (Wan et al., 2022). 1-Metoxyphenazine was synthesized by using methyltransferase LphzM and *S*-adenosyl methionine (SAM) (Figure 5A) while the utilization of *N*-monooxygenase NaphzNO1, NADH and FAD, had yielded 1-hydroxyphenazine *N*′-10-oxide (Figure 5B). Employing these two enzymes in consecutive reactions afforded 1-metoxyphenazine *N*′-10-oxide (Figure 5C), which was detected by HPLC and HRMS (Wan et al., 2022). All of the reactions were carried out in HEPES buffer at pH 7.5 at 30 °C for 3 h. The structures of 1-metoxyphenazine and 1-metoxyphenazine *N*′-10-oxide were confirmed by HRMS and NMR. All reaction products exhibit an even greater potential than 1-hydroxyphenazine to be used as biopesticides (Wan et al., 2022). The same three 1-hydroxyphenazine derivatives were observed as products of biosynthesized in *Pseudomonas chloroplastis* H18 (Wan et al., 2022).

Other reported reactions include mononitration of 1-hydroxyphenazine upon exposure to myeloperoxidase in the presence of nitrates on an analytical scale (Reszka et al., 2012), but the exact structure of the product remains unknown, as it was only detected spectrophotometrically and by MS (Reszka et al., 2012). Other transferases or lipases, and vinyl esters or anhydrides as acyl donors, could yield novel bioactive esters, while the planar phenazine moiety could undergo biocatalytic halogenation (Figure 1Eiii).

### 6.4 Pyoverdine

Pyoverdine (Figure 1Eiv) is a siderophore produced by fluorescent strains of *P. aeruginosa* known for its potential medical and agricultural application, but regardless of these biological activities, there have been no publications regarding its structural optimization via late-stage biocatalysis. Outside of the peptide backbone, amino groups could undergo alkylation by transferases and the carboxylic group could undergo esterification or reduction. In addition, the guanidinium group cold also undergo reduction while other groups in the peptide backbone containing ester and amide bonds could be possible substrates for different hydrolyses (Figure 1Eiv).

Several other natural products from *Pseudomonas* spp., such as aeruginosin A (pyorubin), aeruginosin B and pyomelanin were researched, but no biocatalytic reactions were found reported for these molecules.

## 7 Discussion and perspectives

BNPs still hold a pivotal position in contemporary medicine due to their efficacy and broad applications, which is also reflected in their wide clinical use. However, some BNPs also display drawbacks that hinder their progress as potential therapeutics. When separated from their producing organisms, they can often exhibit suboptimal pharmacological characteristics, such as high toxicity, unfavorable pharmacokinetics, low solubility and chemical instability. These limitations may be partially responsible for the ongoing shift away from the exploration, investigation, and advancement of natural product-based drug discovery. Late-stage functionalization of these compounds holds promise for efficient structural optimizations. However, it is often a challenging task when small and precise modifications must be made on complex natural structures.

In today’s world of synthetic chemistry, enzyme catalysis (biocatalysis) has emerged as a key discipline to enable structural optimizations under milder conditions. This is primarily driven by the remarkable advancements in the biocatalysis field facilitated by bioinformatics and enzyme engineering. With these tools, scientists have unlocked a vast repository of biocatalysts, offering an astonishing array of selective reactions for the effective diversification of various complex natural scaffolds. This approach not only enables high reaction selectivity and chirality control, but is also regarded as an environmentally friendly, sustainable and cost-effective technique, opening the doors for solutions in drug development.

While biocatalysis does offer advantages compared to chemical derivatization, it has some drawbacks, *e.g.*, potential enzyme instability, susceptibility to substrate or product inhibition, insufficient number of well-characterized and ready-to-use biocatalysts. However, modern genomics approaches, which utilize sequence-similarity-based bioinformatics strategies, help to unlock the untapped potential of biosynthetic gene cluster of bacteria to reveal the biosynthesis of natural products (Bommarius, 2015; Scott and Piel, 2019; Romero et al., 2023). These approaches allow us to explore new avenues and overcome some of the challenges associated with biocatalysts, *e.g.*, getting them thoroughly characterized, thus making them susceptible for further design and engineering. Another example of getting sequence-function insights is the family-wide profiling, which was successfully employed to acquire knowledge on flavin-dependent halogenases whose chemical reactivity can differ significantly depending on the producing strain. These profiling efforts have determined previously unknown data on their binding sites, halide specificity, regioselectivity and substrate specificity. Novel and unlikely substrate scopes were unveiled, especially related to large, three-dimensionally complex compounds, a task which would previously require several rounds of directed evolution to accomplish (Fisher et al., 2019; Jeon et al., 2021). Additional evidence in favor of sequence-function correlation is an *in vitro* prototyping workflow for screening a library of *O*-methyltransferase enzymes (Haslinger et al., 2021). While genome sequence databases contain thousands of putative *O*-methyltransferases and keep expanding, only a limited number have undergone detailed functional studies. To address this, a library of *O*-methyltransferase were screened against a range of substrates in just 3 days, allowing to rapidly prototype many uncharacterized *O*-methyltransferases for future use as biocatalysts, as well as to understand their sequence-function correlation.

There is also substantial potential to make new derivatives of complex natural structures like polyketides by swapping domains in gigantic multi-enzyme type I polyketide synthases (PKS) (Kalkreuter and Williams, 2018; Bozhüyük et al., 2019), nonribosomal peptides (NRPs) (Kries et al., 2015; Calcott et al., 2020), hybrid PKS-NRPS (Simunovic et al., 2006) or others, which play crucial roles in their synthesis. This strategy facilitated the accelerated evolution of modular PKS genes, including deletion, addition, or replacement of multiple modules, enabling creation of a diversity orientated library of new derivatives (Wlodek et al., 2017; Nava et al., 2023). The synthetic biology of modular proteins using methods that allow full combinatorial, complete homology-independent shuffling from an unlimited number of parental genes holds promise for the production of valuable chemical entities based on natural products (Maervoet and Briers, 2017; Qian et al., 2020).

In the future, enzymes being discovered from the *Streptomyces* genus might hold particular importance, given that it's the most profilic genus in BNP production. A number of laccases and transaminases showing exceptional thermostability and affinity for branched ketones, dicarbonyl compounds, aldehydes and keto acids were described from *Streptomyces* spp. (Ferrandi et al., 2021). Thus, their potential for the late-stage diversifications of BNPs should also be explored. The overview of late-stage diversifications of the selection of 21 BNPs revealed a number of their chemical derivatizations, but highlighted some important enzymatic transformations. It can be anticipated that structural optimizations via enzymatic and whole-cell biocatalysis will take a prominent role in drug development efforts in the years to come.
