# Supplementary material for: Late-stage diversification of bacterial natural products through biocatalysis
Source: Front Bioeng Biotechnol. 2024 May 14;12:1351583. doi: 10.3389/fbioe.2024.1351583 (PMC11130421; doi:10.3389/fbioe.2024.1351583)
Supplement: Supplementary file 1 [file DataSheet1.docx]

Supplementary Material

Late-stage diversification of bacterial natural products through biocatalysis

Jelena Lazic^1^*, Vuk Filipovic^1^, Lena Pantelic^1^, Jelena Milovanovic^1^, Sandra Vojnovic^1^, Jasmina Nikodinovic-Runic^1^*

^1^ Institute of Molecular Genetics and Genetic Engineering, University of Belgrade, Belgrade, Serbia

* Correspondence:
Jelena Lazic
[jelena_lazic@imgge.bg.ac.rs](mailto:jelena_lazic@imgge.bg.ac.rs)

Jasmina Nikodinovic-Runic
[jasmina.nikodinovic@imgge.bg.ac.rs](mailto:jasmina.nikodinovic@imgge.bg.ac.rs)

**TABLE S1 Late stage diversifications of selected bacterial natural products**

| **Row (Ref.)** | **Bacterial natural product (Substrate)** | **Biocatalyst and reaction conditions** | **Product(s)** |
| --- | --- | --- | --- |
| i  (O'Hara et al., 1988) |   **Streptomycin** | *Bacillus subtilis* crude extract |  |
| ii  (Shang et al., 2016) |   **Tetracycline**: R_1_ = H, R_2_ = Me, R_3_ = OH  **Minocycline**: R_1_ = NMe_2_, R_2_ = H, R_3_ = H  **Chlortetracycline**: R_1_ = Cl, R_2_ = Me, R_3_ = OH | *Paecilomyces* sp. PYG agar 26.5 °C |   **Seco-cyclines** |
| iii  (Shang et al., 2016) |   **Oxytetracycline**: R = OH  **Doxycycline**: R = H | *Paecilomyces* sp. PYG agar 26.5 °C |   **Hemi-cyclines** |
| iv  (Nie et al., 2021) |   **Unconventional tetracycline** | a) TjhO5, 30 °C, Tris-HCl pH 8, NADPH  b) TjhD4, 30 °C, Tris-HCl pH 8, NADH |  |
| v  (Decker et al., 1991) |   **Nikkomycin Z** | Bromoperoxidase from *Streptomyces aureofaciens* Tü 24, buffer 1 M NaOAc, 91 mM NaBr, 7 mM H_2_O_2_, pH 5.5 |  |
| vi  (Decker et al., 1991) |   **Nikkomycin Z** | Bromoperoxidase from *S. aureofaciens* Tü 24, buffer 1 M NaOAc, 91 mM NaBr, 7 mM H_2_O_2_, pH 5.5 |  |
| vii  (Bongs and van Pée, 1994) |   **Pyrrolnitrin** | Non-heme haloperoxidases from *Pseudomonas pyrrocinia* and *Streptomyces aureofaciens* Tü24, Buffer 10 mM NaCl, 7.2 mM H_2_O_2_, pH 5.5 |  |
| viii  (Katzenmeyer et al., 2010) |   **Doxorubicin** | Post mitochondrial fraction prepared from Fischer 344 rat liver, MgCl_2_ (5 mM), NADP (0.25 mM), glucose-6-phosphate (2.5 mM), and doxorubicin (50 μM) incubated at 37 °C |   **7-Deoxydoxorubicin aglycone** |
| ix  (Katzenmeyer et al., 2010) |   **Doxorubicin** | PMF prepared from Fischer 344 rat liver, MgCl_2_ (5 mM), NADP (0.25 mM), glucose-6-phosphate (2.5 mM), and doxorubicin (50 μM) incubated  at 37 °C |   **Doxorubicinol** **7-Deoxydoxorubicinol**  **aglycone** |
| x  (Piska et al., 2021) |   **Daunorubicin** | Human liver cytosol by carbonyl reductase 1 (CBR1) and aldo-keto reductase 1C3 (AKR1C3) |   **Daunorubicinol** |
| xi  (González-Sabín et al., 2011) |   **FK506** | 1. CAL-B, bromoester, TBME, 40 °C  2. Thiol terminated poly(ethylene glycol), Pr_2_NEt/MeCN |  |
| xii  (Na et al., 2016) |   **FK506** | Glucosyltransferase |   **FK506-G** |
| xiii  (Na et al., 2016) |   **FK506** | Sialyltransferase |   **FK506-S** |
| xiv  (Ho et al., 2021) | ****  **Pyochelin** | *Phellinus noxius* |   **Pyochelin-GA** |
| xv  (Jenul et al., 2023) | ****  **Pyochelin** | *S. aureus* pyochelin methyltransferase (Spm) |   **Pyochelin methyl ester** |
| xvi  (Reszka et al., 2004) | ****  **Pyocyanin** | Microperoxidase 11 (MP11), H_2_O_2_ in 50 mM phosphate buffer, pH 6.0, 30 min | **** |
| xvii  (Reszka et al., 2012) | ****  **Pyocyanin** | Lactoperoxidase (0.2 µM), NaNO_2_ (5.8 mM), H_2_O_2_ (5.9 mM), Na-acetate buffer pH 5.2  or  Myeloperoxidase (0.2 U/mL), NaNO_2_ (1 mM), H_2_O_2_ (1 mM), Na-acetate buffer pH 5 |  |
| xviii  (Reszka et al., 2012) | ****  **Pyocyanin** | Myeloperoxidase (1 U/mL), NaNO_2_ (3 mM) NaCl (150 mM) H_2_O_2_ (3 mM), Sodium acetate buffer pH 5 | monochlorinated isomers of pyocyanin, dichloropyocyanin,  small amounts of two dibromoisomers of pyocyanin  and a mixed bromochloropyocyanin |
| xix  (Costa et al., 2017) | ****  **Pyocyanin** | Tautomerizing demethylase from *Mycobacterium fortuitum* (PodA) |   **1-Hydroxyphenazine, formaldehyde** |

**References**

Bongs, G., and Van Pée, K.-H. (1994). Enzymatic chlorination using bacterial nonheme haloperoxidases. *Enzyme Microb Technol* 16**,** 53-60.

Costa, K.C., Glasser, N.R., Conway, S.J., and Newman, D.K. (2017). Pyocyanin degradation by a tautomerizing demethylase inhibits *Pseudomonas aeruginosa* biofilms. *Science* 355**,** 170-173.

Decker, H., Pfefferle, U., Bormann, C., Zähner, H., Fiedler, H.P., Van Pée, K.H., Rieck, M., and König, W.A. (1991). Metabolic products of microorganisms. 258. Enzymatic bromination of nikkomycin Z. *J Antibiot (Tokyo)* 44**,** 626-634.

González-Sabín, J., Morán-Ramallal, R., and Rebolledo, F. (2011). Regioselective enzymatic acylation of complex natural products: Expanding molecular diversity. *Chem Soc Rev* 40**,** 5321-5335.

Ho, Y.-N., Hoo, S.Y., Wang, B.-W., Hsieh, C.-T., Lin, C.-C., Sun, C.-H., Peng, C.-C., Lin, C., and Yang, Y.-L. (2021). Specific inactivation of an antifungal bacterial siderophore by a fungal plant pathogen. *ISME J* 15**,** 1858-1861.

Jenul, C., Keim, K.C., Jens, J.N., Zeiler, M.J., Schilcher, K., Schurr, M.J., Melander, C., Phelan, V.V., and Horswill, A.R. (2023). Pyochelin biotransformation by *Staphylococcus aureus* shapes bacterial competition with *Pseudomonas aeruginosa* in polymicrobial infections. *Cell Rep* 42**,** 112540.

Katzenmeyer, J.B., Eddy, C.V., and Arriaga, E.A. (2010). Tandem laser-induced fluorescence and mass spectrometry detection for high-performance liquid chromatography analysis of the in vitro metabolism of doxorubicin. *Anal Chem* 82**,** 8113-8120.

Na, Y.G., Jun, H.S., Kim, D., Park, B.C., Lim, S.K., Lee, K.H., Hwang, S.J., Park, J.S., Jung, S.H., and Cho, C.W. (2016). Preformulation of FK506 prodrugs for improving solubility. *Bull Korean Chem Soc* 37**,** 1313-1319.

Nie, Q.-Y., Ji, Z.-Y., Hu, Y., and Tang, G.-L. (2021). Characterization of highly reductive modification of tetracycline D-ring reveals enzymatic conversion of enone to alkane. *ACS Catal* 11**,** 8399-8406.

O'hara, K., Ohmiya, K., and Kono, M. (1988). Structure of adenylylated streptomycin synthesized enzymatically by *Bacillus subtilis*. *Antimicrob Agents Chemother* 32**,** 949-950.

Piska, K., Jamrozik, M., Koczurkiewicz-Adamczyk, P., Bucki, A., Żmudzki, P., Kołaczkowski, M., and Pękala, E. (2021). Carbonyl reduction pathway in hepatic in vitro metabolism of anthracyclines: Impact of structure on biotransformation rate. *Toxicol Lett* 342**,** 50-57.

Reszka, K.J., O'malley, Y., Mccormick, M.L., Denning, G.M., and Britigan, B.E. (2004). Oxidation of pyocyanin, a cytotoxic product from *Pseudomonas aeruginosa*, by microperoxidase 11 and hydrogen peroxide. *Free Radic Biol Med* 36**,** 1448-1459.

Reszka, K.J., Xiong, Y., Sallans, L., Pasula, R., Olakanmi, O., Hassett, D.J., and Britigan, B.E. (2012). Inactivation of the potent *Pseudomonas aeruginosa* cytotoxin pyocyanin by airway peroxidases and nitrite. *Am J Physiol Lung Cell Mol Physiol* 302**,** L1044-L1056.

Shang, Z., Salim, A.A., Khalil, Z., Bernhardt, P.V., and Capon, R.J. (2016). Fungal biotransformation of tetracycline antibiotics. *J Org Chem* 81**,** 6186-6194.
